# Probiotic biofilm modified bioceramics for bone defect healing via osteogenesis, angiogenesis, and immune modulation

**DOI:** 10.3389/fphar.2025.1588023

**Published:** 2025-05-13

**Authors:** Junwei Su, Huiyun Gu, Xiang Huang, Ying Yuan, Yunchang Zhao, Fan Yang, Yong Zhao

**Affiliations:** Department of Orthopedic Trauma and Microsurgery, Zhongnan Hospital of Wuhan University, Wuhan, Hubei, China

**Keywords:** bone tissue engineering, immunomodulation, Wnt/β-catenin, probiotic biofilm, *Lactobacillus* acidophilus

## Abstract

The failure to repair bone defects in a timely manner has a detrimental effect on patients’ quality of life and functional status. Consequently, there are increasing demands for medical interventions to promote healing of bone defects. However, the local inflammation induced by implants and the side effects associated with the systemic use of drugs have prompted research into the development of bioactive materials. Recent reports have indicated that oral administration of *Lactobacillus acidophilus* (LA) can act as an immunomodulator. In this study, we have strategically designed bioceramic scaffolds modified with inactivated LA biofilms (LA@BC) through UV irradiation for localized application of LA. The biosafety of the scaffold was validated at the cellular and animal levels to ensure that it can be safely used without bacteraemia. LA@BC achieved M1 to M2 polarization of macrophages *in vitro* by reducing the secretion of inflammatory factors. In addition, LA@BC enhanced the osteogenic effect of bone marrow mesenchymal stem cells by modulating the Wnt/β-catenin signaling pathway. Furthermore, osteogenesis and angiogenesis complement each other. LA@BC exerted a positive effect on the angiogenic effect of endothelial cells. In a rat cranial defect model, LA@BC upregulated the expression of RUNX2, OCN, CD31, and IL-10 in tissues, again demonstrating potent immunomodulatory and osteogenic effects. In conclusion, this bioactive scaffold provides a new strategy for clinical bone repair.

## 1 Introduction

Bone defects have significant public health and socio-economic ramifications for patients (Shen et al., 2024). The limited regenerative capacity of bone tissue necessitates complex and demanding treatment for bone defects resulting from severe trauma, tumor resection, and congenital malformations (Fan et al., 2024). Bone regeneration through classical autologous or allogeneic bone grafting strategies still suffers from clinical limitations, including scarcity of bone donors, insufficient osteogenic potential, pathogenic infections and immunogenic rejection (Miron et al., 2024). In recent years, synthetic bone graft development has become a significant research focus (Hu et al., 2024; Liu et al., 2023). Scaffolds based on calcium phosphate and calcium sulphate have been gradually applied to fill therapeutic scenarios with insufficient grafted bone (Xu et al., 2024). However, the utilization of synthetic bone materials typically results in local inflammation, which in turn disrupts the normal microenvironment and hinders osseointegration (Zhao et al., 2024).

The focus on microbiologically mediated therapies has garnered considerable interest (Su et al., 2025; Gu et al., 2024). Probiotics, defined as non-pathogenic bacteria, have been shown to inhibit the growth of potentially harmful bacteria and to regulate the body’s microenvironment. *Lactobacillus* acidophilus (LA) is a probiotic bacterium that is naturally present in the human gut, where it has been shown to inhibit the adhesion of potentially harmful bacteria to intestinal cells (Goh et al., 2021). Oral LA has been demonstrated to modulate the human immune system, resulting in its widespread utilization in the treatment of various immune-related diseases, including rheumatoid arthritis and ulcerative colitis (Bungau et al., 2021; Aximujiang et al., 2022). In previous studies, LA barely activated macrophage inflammatory responses and upregulated inflammatory factors compared to *Lactobacillus* salivarius and *Lactobacillus* royale, suggesting that LA may have an advantage in modulating the immune system (Quinteiro-Filho et al., 2015). However, the use of LA in the treatment of bone defects remains largely unexplored, especially since its co-existence with living animals has not been clearly defined.

It is well established that bacteria have the capacity to adhere to implants over extended periods, leading to the persistence of infections (Montanaro et al., 2007; Arciola et al., 2018). In an effort to circumvent these challenges, a novel approach was devised, entailing the modification of LA in the mode of bacterial biofilm to bioceramic scaffolds (LA@BC). This modification was undertaken to ascertain its capacity in promoting bone repair. Notably, the probiotic properties of LA@BC were retained even after inactivation, thereby circumventing biosafety concerns typically associated with live bacteria. The anti-inflammatory, osteogenic, and angiogenic capacities of LA@BC were verified both *in vitro* and *in vivo*. Consequently, probiotic biofilms formed on implants have the potential to become a novel biomaterial for the treatment of a wide range of bone defects.

## 2 Materials and methods

### 2.1 Preparation of bioceramics

Bioceramic (BC) scaffolds were developed based on prior research involving tricalcium phosphate (TCP). Synthetic β-Ca_3_(PO_4_)_2_ (β-TCP) powder was generated through an aqueous precipitation method, utilizing a solution of diammonium phosphate (NH_4_)_2_HPO_4_) and calcium nitrate (Ca(NO_3_)_2_·4H_2_O) (Bouchart et al., 2020; Li M. et al., 2021). The β-TCP powder was then mixed with polyvinyl alcohol and placed into a circular mold, followed by sintering at 1,150°C under controlled pressure. The resulting scaffolds were cleaned and dried before undergoing a final sintering process at 1,150°C to create the bioceramic scaffolds.

### 2.2 Culture of probiotics

*Lactobacillus* acidophilus (LA, ATCC 13651) was purchased from the Guangdong Microbial Cultural Collection Center (GDMCC). LA was cultured with MRS broth at 37°C. The LA bacterial solution was authenticated by strain identification for subsequent use.

### 2.3 Preparation of LA biofilm modified BC

The BC scaffold was cultured with LA solution to obtain probiotic biofilm modified BC(L@BC). Briefly, 100 μL of freeze-dried solution from a LA single colony was added to 10 mL of MRS (de Man, Rogosa and Sharpe) broth to culture for 24 h. The BC subjected to ultrasonic cleaning, drying and ultraviolet ray treatment was cultured with LA in 24-well plates. After 24 h of coculture, the BC was gently washed with sterilized PBS for three times to remove planktonic bacteria.

### 2.4 Morphological observation of LA biofilm

LA was cultured on BC scaffold for 24 h. Excess LA was washed away with PBS, and the cells were fixed in 2.5% glutaraldehyde for 2 h. The cells were then gradient-dehydrated with 30%, 50%, 70%, 90% and 100% ethanol for 15 min each. Finally, the cell morphologies of the different samples were observed under a scanning electron microscope (SEM) from TESCAN, Czech Republic. Additionally, an energy dispersive spectrometer (EDS) was utilized to investigate the distribution and alteration of elements.

### 2.5 Inactivation of LA biofilm by UV sterilization

LA@BC was stained with SYTO9 and propidium iodide (PI) both before and after UV sterilization at room temperature for 30 min. The staining solution consisted of 1,000 μL of PBS and 1 μL of SYTO9, and 1 μL of PI which was incubated for 30 min at 37°C. Excess SYTO9 and PI was washed away by rinsing the sample three times with PBS. Observations were conducted using laser confocal microscopy. The excitation and emission wavelengths for SYTO9 were 480 and 500 nm, respectively, while those for PI were 535 and 615 nm.

### 2.6 MRS plating method for *Lactobacillus* probiotics

To assess the viability of probiotics after UV sterilization, MRS agar plate method was used. First, LA@BC, both un- and UV-sterilized, were washed in PBS for 1 h at 170 rpm with oscillation. 100 μL of eluted LA bacteria were evenly spread on the surface of MRS agar plates using a sterile applicator. The plates were then incubated anaerobically at 37°C for 24–48 h for optimal growth. At the end of incubation, the MRS plates were observed to visually assess the viability of the *Lactobacillus* strains. The plates were then incubated anaerobically at 37°C for 24–48 h to allow for optimal growth. After incubation, colony-forming units (CFU) were counted to assess the growth and viability of the *Lactobacillus* strains. This method allowed for effective monitoring of LA viability and the evaluation of its characteristics in various formulations.

### 2.7 Cell culture

The rat bone marrow mesenchymal stem cells (BMSCs) were extracted from the bone marrow cavity of 4-week-old Sprague Dawley (SD) rats and co-cultured with α-MEM supplemented with 10% fetal bovine serum (FBS) and 1% penicillin-streptomycin solution. RAW264.7 macrophages and Human umbilical vein endothelial cells (HUVECs) were cultured in Dulbecco’s Modified Eagle Medium (DMEM) supplemented with 10% FBS and 1% penicillin-streptomycin solution. All cells were grown at 37°C in a humidified atmosphere containing 5% CO_2_.

### 2.8 Cytotoxicity of LA@BC

To evaluate the cell proliferation potential of LA@BC, a Cell Counting Kit-8 (CCK-8) assay was conducted. BMSCs or HUVECs were seeded in 24-well plates at a density of 1 × 10^6^ cells per well. Following attachment for 24 h, the cells were treated with BC, LA@BC. Control wells received no treatment. After incubation for 24, 48, and 72 h, 50 µL of CCK-8 solution was added to each well, and the plates were incubated for an additional 2 h at 37°C. The absorbance was measured at 450 nm using a microplate reader, with background subtraction performed at 630 nm. The results were expressed as a percentage of cell proliferation compared to the control group.

### 2.9 Live/dead staining

BMSCs (or HUVECs) were cultured at 1 × 10^5^ cells per well in 24-well plates according to standard methods. Once the cells reached 90% confluence, they were seeded onto the surfaces of BC and LA@BC scaffolds for 24 h (or 48 h) to assess cell viability. A live/dead cell labeling kit (C2015M), which includes propidium iodide (PI, red fluorescence) and Calcein-AM (green fluorescence), was employed to evaluate cell viability. The cells on the scaffolds were stained for 60 min with 500 μL of the combined dye solution. Fluorescence images were captured using an inverted fluorescence microscope (ICX41, SOPTOP, China).

### 2.10 *In vitro* osteogenesis of BMSCs

The third passage BMSCs were seeded in Control, BC and LA@BC cultured in the osteogenic induction medium. It is crucial to monitor the cells, ensuring that the medium is changed every three to 4 days to maintain optimal conditions for proliferation and differentiation.

### 2.11 ALP activity

At day 7 of induction, alkaline phosphatase (ALP) staining is performed to assess early markers of osteogenic differentiation. After culturing the cells for 7 days, we gently washed them with PBS three times. Then, we fixed the cells with 4% paraformaldehyde for 20 min. Next, we applied a BCIP/NBT alkaline phosphatase chromogenic kit (Beyotime, Shanghai, China) for 30 min at room temperature. After staining, the BMSCs were washed with PBS and observed using a microscope (ICX41, SOPTOP, China).

### 2.12 Alizarin Red staining

At day 21, Alizarin Red S staining is conducted to evaluate calcium deposition, which signifies mineralization, an essential aspect of bone formation. After culturing the cells for 21 days, we gently washed the cell medium with PBS three times. Then, we fixed the cells with 4% paraformaldehyde for 20 min, then stained them with Alizarin Red S for 30 min at room temperature. Finally, we rinsed with PBS to remove excess stain. Following the completion of staining, a microscope (ICX41, SOPTOP, China) were taken and microscopic observations were made to evaluate the impact of LA@BC on the osteogenic differentiation of BMSCs.

### 2.13 Quantitative real-time polymerase chain reaction

The expression levels of Wnt/β-catenin pathway genes Wnt, β-catenin (from BMSCs), angiogenesis-related genes Vascular Endothelial Growth Factor (VEGF), basic Fibroblast Growth Factor (bFGF) (from HUVECs), inflammation-related genes tumor necrosis factor-α (TNF-α), interleukin-10 (IL-10) (from RAW264.7 cells), osteogenesis-related genes Wnt, β-catenin, runt-related transcription factor 2 (RUNX2) and osteocalcin (OCN) (from Rat cells) were tested. The GAGDH were respectively used as internal control genes. Table 1 showed the primer sequences of the tested genes.

**TABLE 1:** RT-qPCR primer sequences.

Gene	Forward primer (5′ to 3′)	Reverse primer (5′ to 3′)
Wnt (Wnt3a)	AGT​ATT​CCT​CCC​TGG​GCT​CG	TGC​CAA​TCT​TGA​TGC​CCT​CG
β-catenin	CAT​CTA​CAC​AGT​TTG​ATG​CTG​CT	GCA​GTT​TTG​TCA​GTT​CAG​GGA
RUNX2	CGG​AAT​GCC​TCT​GCT​GTT​AT	TTC​CCG​AGG​TCC​ATC​TAC​TG
OCN	TGC​ATT​CTG​CCT​CTC​TGA​CC	ACC​ACC​TTA​CTG​CCC​TCC​TG
GAGDH (Rat)	GGT​GGA​CCT​CAT​GGC​CTA​CA	CTC​TCT​TGC​TCT​CAG​TAT​CCT​TGC​T
VEGF	CTT​GCC​TTG​CTG​CTC​TAC​CT	GCA​GTA​GCT​GCG​CTG​ATA​GA
bFGF	GCT​GTA​CTG​CAA​AAA​CGG​GG	AGC​CAG​GTA​ACG​GTT​AGC​AC
GAGDH (Human)	GGA​AGC​TTG​TCA​TCA​ATG​GAA​ATC	TGA​TGA​CCC​TTT​TGG​CTC​CC
TNF-α	CGC​TCT​TCT​GTC​TAC​TGA​ACT​TCG​G	GTG​GTT​TGT​GAG​TGT​GAG​GGT​CTG
IL-10	GCT​CTT​ACT​GAC​TGG​CAT​GAG	CGC​AGC​TCT​AGG​AGC​ATG​TG
GAGDH (Mouse)	CCT​CGT​CCC​GTA​GAC​AAA​ATG	TGA​GGT​CAA​TGA​AGG​GGT​CGT

### 2.14 Migration assay

HUVECs were plated at a density of 1 × 10^6^ cells per well in 6-well plates. Once the cells reached 90% confluency, a linear scratch was made through the monolayer using a sterile 100-μL pipette tip, and the detached cells were gently washed away with PBS. Transwell inserts containing BC and LA@BC scaffolds were subsequently placed in the wells. The migration of HUVECs into the scratched area was observed at 0, 12, and 24 h. Cells were labeled with Calcein-AM (green fluorescence) and visualized using an inverted fluorescence microscope. The healing ratio of the scratched area was quantified using ImageJ software.

### 2.15 Matrigel tube formation assay

Matrigel matrix was used to coat 24-well plates with 200 µL per well prior to seeding HUVECs at a density of 5 × 10^4^ cells per well. Transwell inserts containing BC and LA@BC scaffolds were carefully placed into the incubated plates. After 6 h, HUVECs were labeled with Calcein-AM (green fluorescence) and observed for tube formation using an inverted fluorescence microscope. Parameters of tube formation were analyzed using ImageJ software.

### 2.16 Enzyme-linked immunosorbent assay

After processing the cells as described above, the supernatants of HUVECs or RAW 264.7 cells were collected to measure cytokines. The secretion of cytokines VEGF, bFGF (from HUVECs), IL-17, IL-4 (from RAW 264.7 cells) was measured by commercial ELISA kits, following the manufacturer’s guidance.

### 2.17 Macrophage response to different scaffolds *in vitro*

LPS (500 ng mL^−1^) stimulated RAW 264.7 after the cells reached 90% confluency. After 12 h, LPS were gently washed with PBS for three times. BC, LA@BC scaffolds were gently transformed into an incubator of the six-well plates. LPS group (regarded as Control group) added PBS of equal capacity. RAW 264.7 cells were co-cultured with different samples for 72 h.

### 2.18 Immunofluorescence staining

As the previous description, RAW 264.7 macrophages were cultured on various samples for a duration of 72 h after stimulation with LPS (500 ng mL^−1^). LPS group (without scaffold) was regarded as Control group. Subsequently, the cells were fixed using 4% paraformaldehyde for 20 min and permeabilized with 0.25% Triton X-100 for 20 min. After permeabilization, the cells were blocked with 1% bovine serum albumin for 1 h to prevent non-specific binding. The cells were then incubated overnight at 4°C with primary antibodies against CD206 and inducible Nitric Oxide Synthase (iNOS) (both sourced from Abcam) at a dilution of 1:100. Following the primary antibody incubation, the cells received a 30-min treatment with secondary antibodies conjugated to either Alexa Fluor 488 or Alexa Fluor 594. Post-incubation, the nuclei were stained for 5 min with 4′,6-diamidino-2-phenylindole (DAPI). The immunofluorescence-stained cells were visualized using a laser scanning confocal microscope (Leica STELLARIS 5 SR, Germany).

### 2.19 *In vivo* cranial bone defect model

The Animal Experimental Ethics Review Committee of Zhongnan Hospital of Wuhan University approved all animal experiments (approval number: ZN2023191), which were conducted in accordance with the National Research Council’s Guidelines for the Care and Use of Experimental Animals. A cranial defect with a diameter of 5.0 mm was created in male rats (Xu et al., 2023). A total of 45 male rats were randomly divided into three groups (n = 15): a control group, a group treated with BC, and a group treated with LA@BC. The implantation of the different biomaterials was then performed. 8 weeks after treatment, all rats in each group were euthanized. Cranial specimens were collected and scanned using micro-CT. The micro-CT data were analyzed using CTan, CTvox, and CTvol software to determine the proportion of newly formed bone. Following this, all samples were fixed in a 10% neutral formalin solution for 1 week and then decalcified with a 17% EDTA solution for 5 weeks before being processed into paraffin sections. Coronal sections were prepared at 4 μm and subjected to histopathological staining using H&E and Masson’s reagent, as well as immunofluorescence for CD31, TNF-α, and IL-10.

### 2.20 Statistical analysis

Each experiment was evaluated using the mean values ± standard deviation from at least three tests. The researchers performing the interventions and analyzing the outcomes were blinded to the group assignments to minimize bias. Data analysis was performed using GraphPad Prism version 9. One-way ANOVA was used to analyze data from multiple groups, and two-tailed Student’s t-test was used to compare two groups. *P*-value of less than 0.05 was considered statistically significant.

## 3 Results

### 3.1 Preparation and characterization of probiotic biofilm modified BC

**FIGURE 1:**
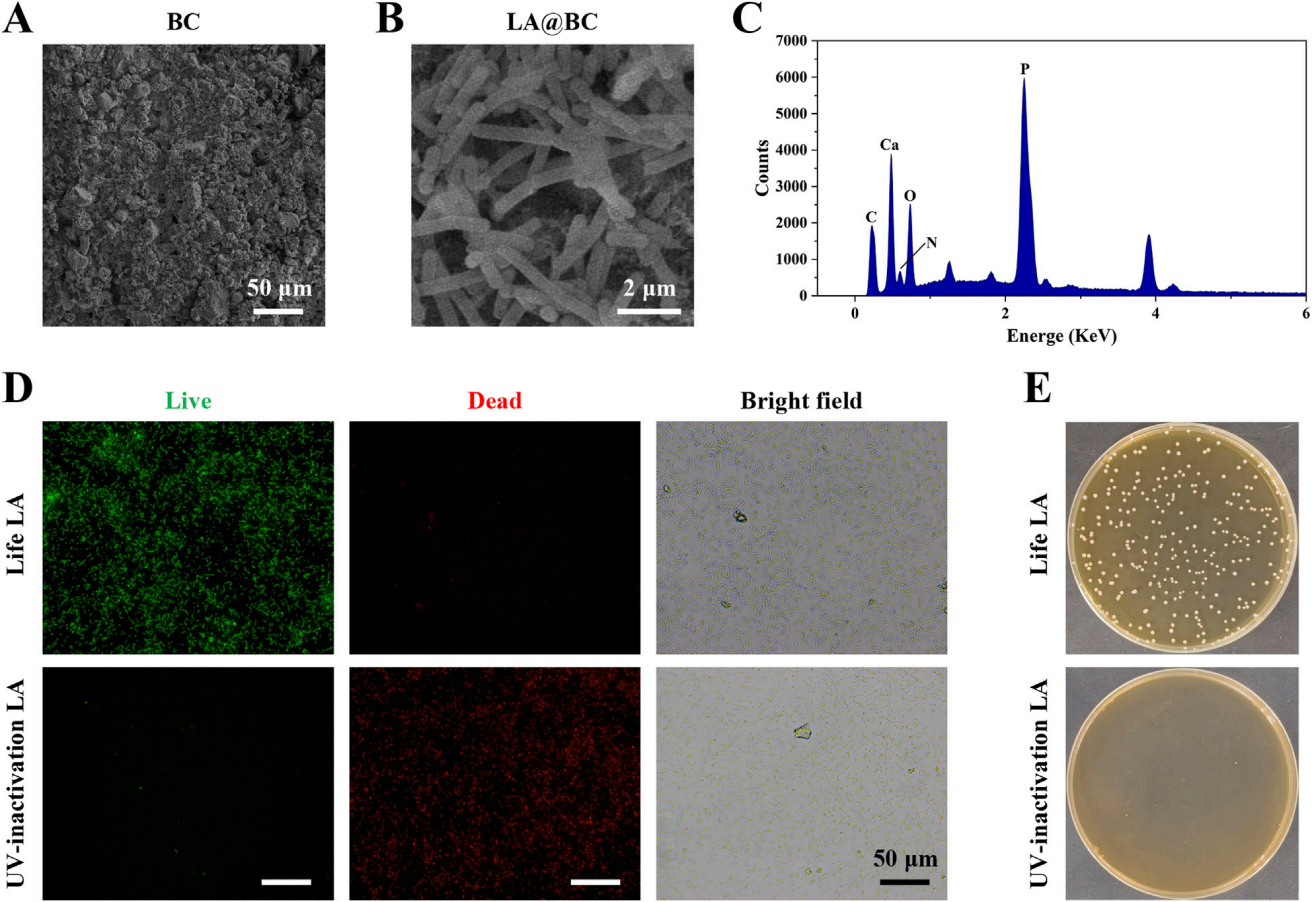
Characterization of LA probiotic biofilm modified BC. **(A)** SEM images of BC. **(B)** SEM images of LA biofilm modified TCP (LA@BC). **(C)** The quantification of elements on LA@BC. **(D)** Live/dead staining for LA on the LA@BC received with or without UV-inactivation. **(E)** LA on MRS agar plates after elution and dilution from the LA@BC received with or without UV-inactivation.

Bioactive ceramics (BC) are frequently utilized in clinical practice for the treatment of bone defects. Nevertheless, the propensity for bacteria to adhere to these implants can result in swift biofilm formation, which complicates the management of osteomyelitis. To improve the efficacy of artificial bone, we have opted to integrate probiotics—bacteria that are non-pathogenic—into our strategy. Probiotics provide improved biosafety and enhance osteogenic properties when compared to non-probiotics, such as *S. aureus*, which pose biosecurity risks and have limited osteogenic potential. We have chosen *Lactobacillus acidophilus*, a well-known probiotic, to enhance the properties of BC bioceramics. Figure 1A illustrates the characterization of LA-modified bioactive ceramics (LA@BC). Scanning electron microscope (SEM) revealed that the surface crystals of pure BC appeared as short rod-like structures, accompanied by irregularly distributed porous honeycomb patterns. After LA modification, SEM images displayed a complex hierarchical architecture, featuring intertwined bacterial filaments and extracellular polymeric substances (EPS) that form a three-dimensional biofilm matrix on BC substrates (Figure 1B). Energy-dispersive X-ray spectroscopy (EDS) analysis confirmed the elemental composition of LA@BC, revealing significant peaks for carbon (C), calcium (Ca), nitrogen (N), oxygen (O), and phosphorus (P) (Figure 1C). The presence of the nitrogen peak in LA@BC indicates that the probiotics have been successfully incorporated into the BC matrix. Live/dead staining showed that viable LA cells (green fluorescence) were abundant on the surface of the non-UV-inactivated LA@BC, while dead cells (red fluorescence) were nearly absent. Notably, after UV inactivation, the green fluorescent cells were almost completely eliminated, and red fluorescent cells filled the field of view, indicating successful inactivation of LA (Figure 1D). After plating the eluted LA on MRS agar plates, it was found that the UV-inactivated LA@BC exhibited a significant reduction in colony units on the MRS agar plates compared to the untreated samples, indicating that UV exposure effectively inactivated the bacterial cells (Figure 1E).

### 3.2 LA@BC modulated osteogenic differentiation of BMSCs *in vitro*

**FIGURE 2:**
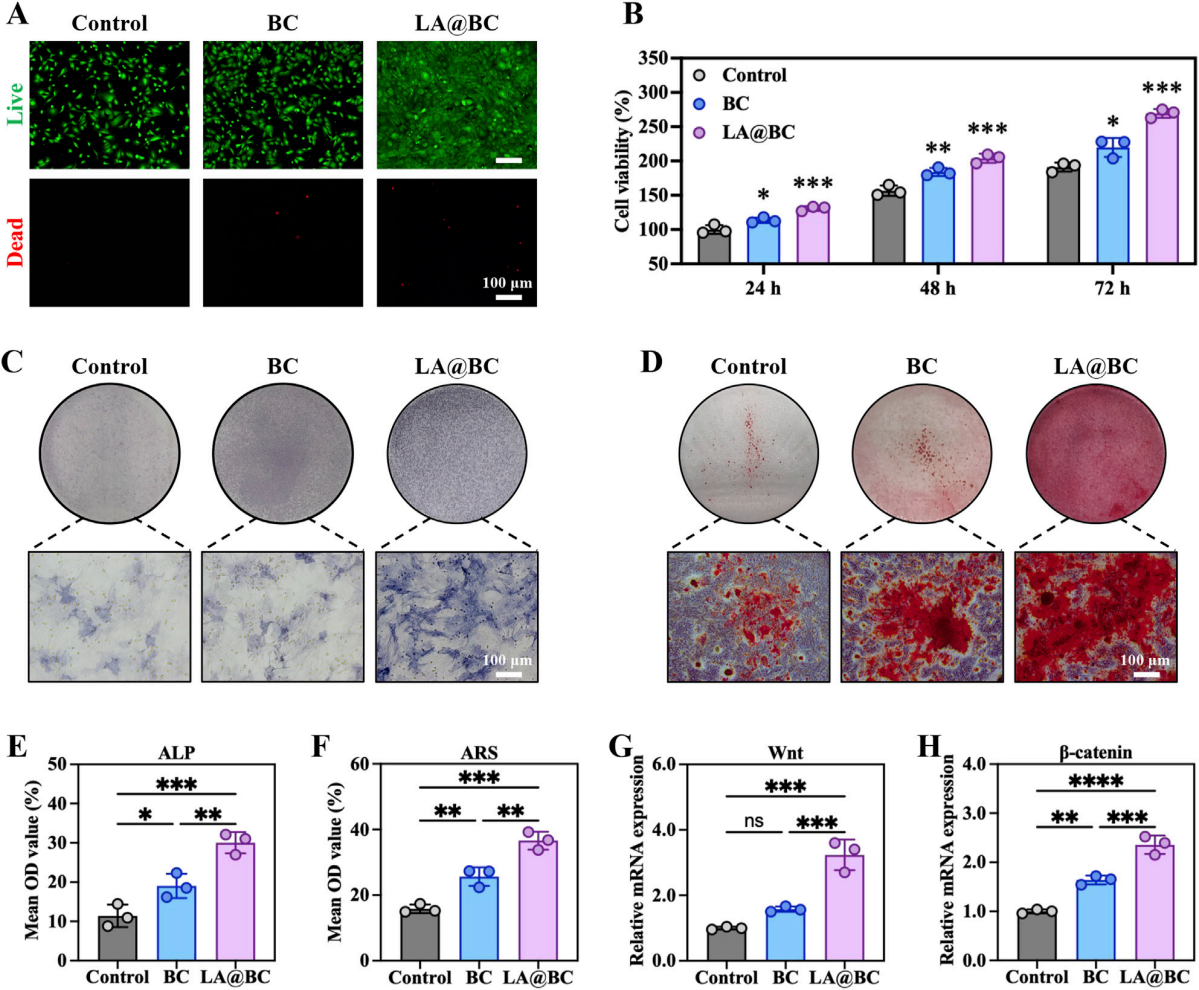
LA@BC promoted BMSCs Osteogenic differentiation *in vitro*. **(A)** Live (green)/dead (red) staining of BMSCs after 24 h. **(B)** Cell proliferation of BMSCs cultured with BC, LA@BC for 24, 48 and 72 h **(C)** ALP staining of BMSCs after 7 days. **(D)** Alizarin Red S staining of BMSCs after 21 days. **(E, F)** Quantitative analysis of ALP staining, and Alizarin Red S staining. **(G, H)** Relative mRNA expression of Wnt, and β-catenin of BMSCs on day 7. *P < 0.05, **P < 0.01, ***P < 0.001, ****P < 0.0001.

For effective treatment of bone defects, it is essential that the materials used possess strong osteogenic properties to promote bone regeneration and prevent complications such as implant failure, multiple surgeries, and bone nonunion. To evaluate the osteogenic potential of LA@BC, we investigated its effects on BMSCs viability, proliferation, and differentiation. Figure 2A showed live/dead cell staining after 24 h, revealing that LA@BC treatment maintained high cell viability (green fluorescence) with minimal apoptosis (red fluorescence), comparable to the control group. Cell viability testing demonstrated that LA@BC significantly enhanced BMSCs proliferation over 72 h, with OD values reaching 1.5-fold of the control group at 72 h (Figure 2B). Following 7 days of osteogenic induction, alkaline phosphatase (ALP) staining exhibited intense ALP (blue) distribution in the LA@BC group, indicating robust early osteogenic differentiation (Figures 2C, E). Similarly, Alizarin Red S staining after 21 days revealed dense mineral deposits (red) in the LA@BC group, which was quantitatively confirmed by higher OD values (Figures 2D, F). The Wnt/β-catenin signaling pathway is a crucial regulatory mechanism in osteogenesis, playing a vital role in the differentiation of BMSCs into osteoblasts and the subsequent formation of bone tissue (Bai et al., 2021). At the molecular level, qRT-PCR analysis revealed that LA@BC upregulated Wnt/β-catenin signaling pathways, with nearly 3-fold and 2-fold respectively increases in Wnt and β-catenin mRNA expression compared to the control (Figures 2G, H). Therefore, these results demonstrated that LA probiotics biofilm effectively enhanced BMSCs osteogenesis *in vitro* through both proliferation acceleration and Wnt/β-catenin pathway activation, providing fundamental support for *in vivo* experiments.

### 3.3 LA@BC enhanced cell proliferation and angiogenesis *in vitro*

**FIGURE 3:**
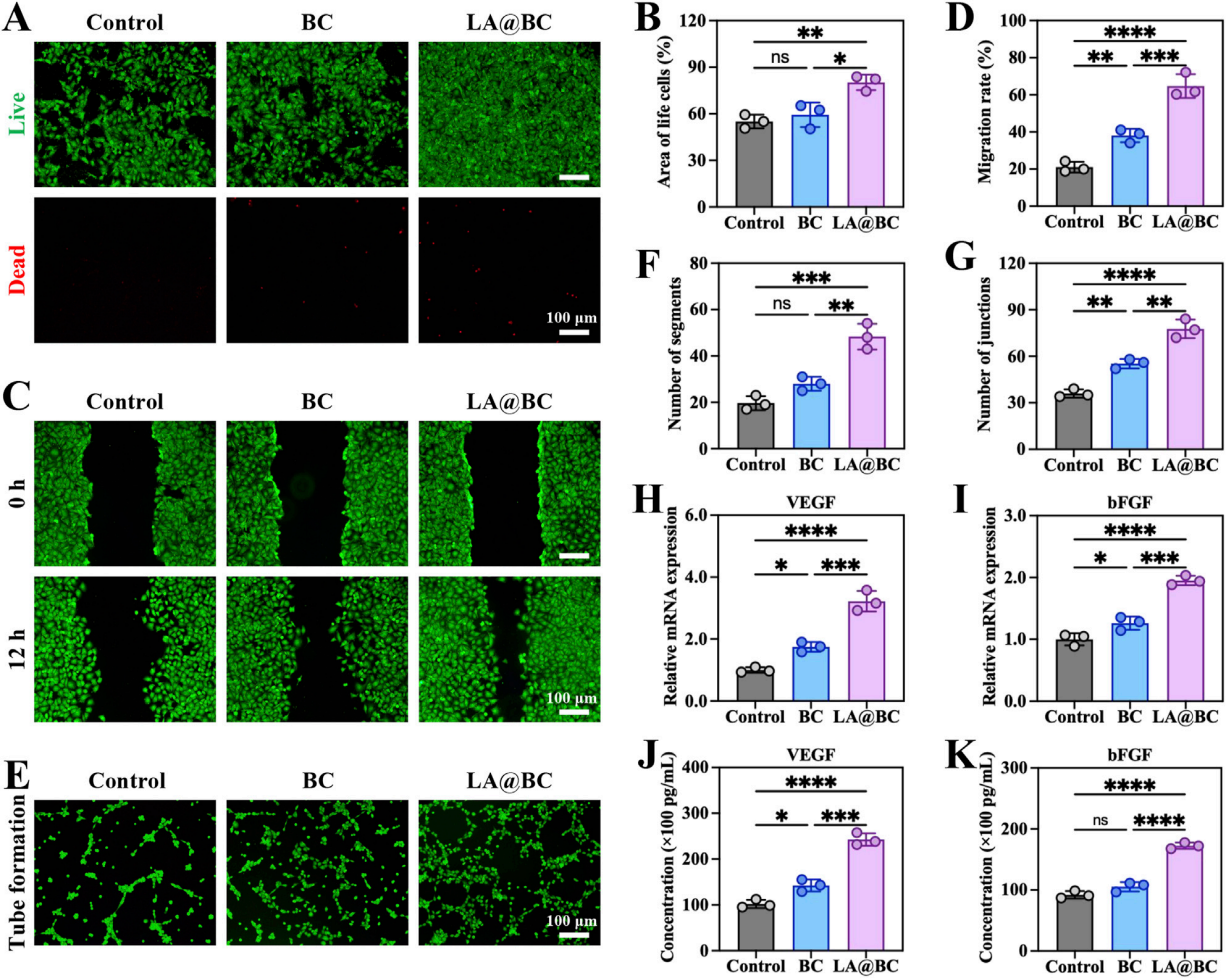
LA@BC promoted HUVECs proliferation and angiogenesis *in vitro*. **(A)** Live (green)/dead (red) staining of HUVECs after 48 h. **(B)** Quantitative area analysis of the living cells based on live/dead staining. **(C)** Scratch wound healing tests on the migration of HUVECs cultured with the LA@BC from 0 to 12 h. **(D)** Quantification of cell migration rate based on scratch wound healing tests. **(E)** Tube formation tests of HUVECs cultured with the LA@BC after 12 h **(F, G)** Quantification of number of segments and junctions based on tube formation tests. **(H, I)** Relative mRNA expression of VEGF, and bFGF on day 7. **(J, K)** ELISA analyses of cytokines VEGF, and bFGF on day 7. *P < 0.05, **P < 0.01, ***P < 0.001, ****P < 0.0001.

Angiogenesis plays a crucial role in promoting osteogenesis by ensuring adequate blood supply and nutrient delivery to bone tissues (Zhou et al., 2021). To investigate the angiogenic potential of LA@BC, we evaluated its effects on HUVEC proliferation, migration, and tube formation. Figure 3A shows that LA@BC treatment maintained high cell viability (green fluorescence) with minimal apoptosis (red fluorescence) after 48 h, suggesting biocompatibility comparable to the control group. Quantitative analysis of the live cell area confirmed that LA@BC enhanced HUVEC proliferation, which is critical for endothelial expansion during angiogenesis (Figure 3B). As illustrated in Figure 3C, the LA@BC group exhibited accelerated wound closure as determined by scratch assays, with near closure observed at 12 h. This rapid migration was further validated by Quantitative analysis, where LA@BC-treated cells exhibited significantly higher migratory capacity (Figure 3D). Tube formation assays revealed that LA@BC induced robust vascular network formation, evidenced by dense tubular structures with numerous segments and junctions (Figures 3E–G). At the molecular level, RT-PCR revealed significant upregulation of VEGF and bFGF mRNA in the LA@BC group, which are key regulators of endothelial function and vascular sprouting (Figures 3H, I). ELISA analysis further demonstrated elevated secretion of VEGF and bFGF proteins (Figures 3J, K), indicating the activation of angiogenic pathways. Therefore, the angiogenic capacity of LA@BC scaffold rendered it more advantageous in osteogenesis.

### 3.4 Anti-inflammatory effects of LA@BC *in vitro*

**FIGURE 4:**
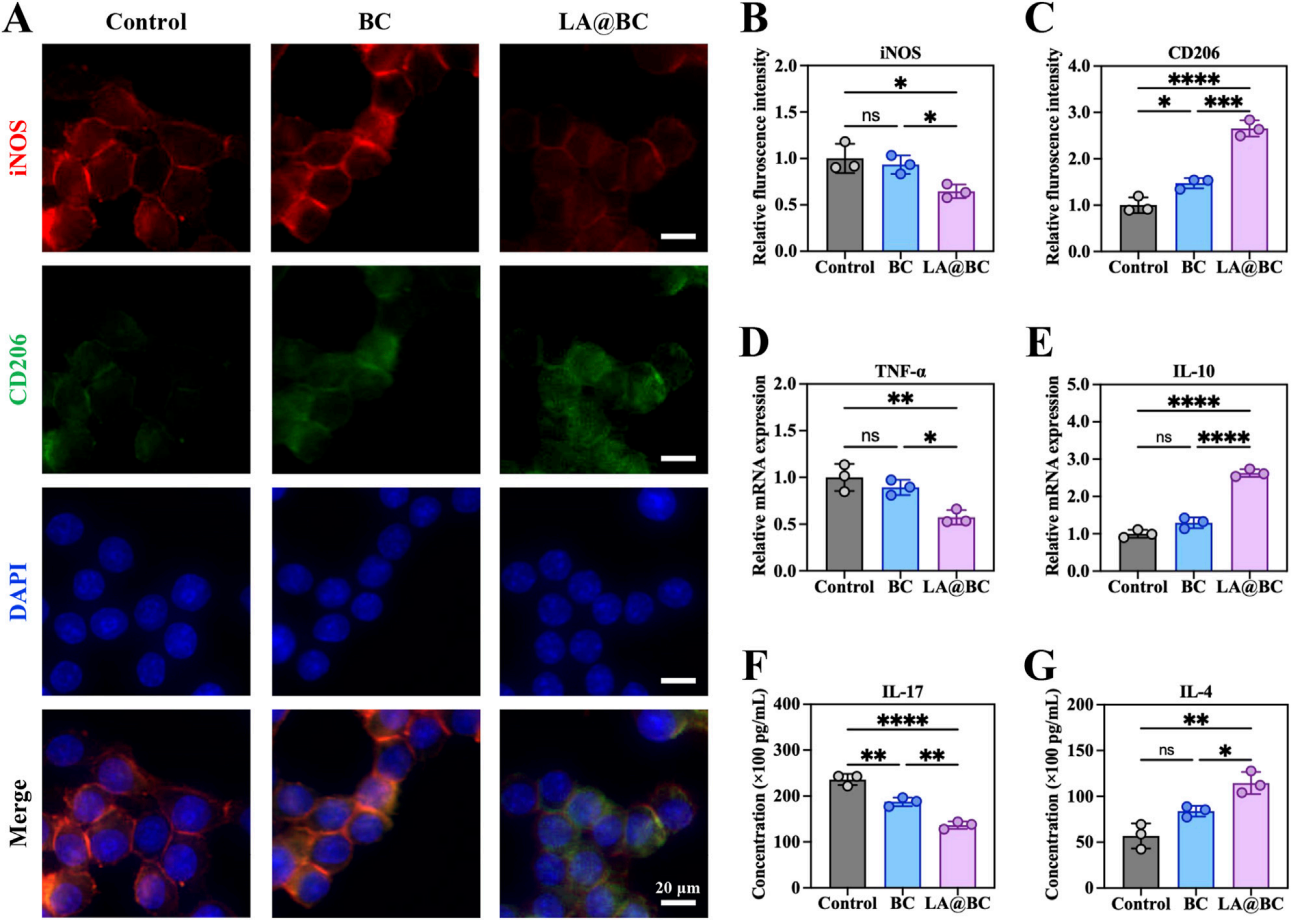
LA@BC regulated macrophage inflammation and polarization *in vitro*. **(A)** Immunofluorescent staining of iNOS (red), CD206 (green) and nucleus (blue) of RAW264.7 cells after 3 days. **(B, C)** Quantitative fluorescence intensity of iNOS and CD206 of RAW264.7 cells based on immunofluorescent images. **(D, E)** Relative mRNA expression of TNF-α, and IL-10 of RAW264.7 cells after 3 days. **(F, G)** ELISA analyses of pro-inflammatory cytokines IL-17, and anti-inflammatory cytokines IL-4 from RAW264.7 cells (after LPS stimulation) treated with the LA@BC after 3 days. *P < 0.05, **P < 0.01, ***P < 0.001, ****P < 0.0001.

Macrophages play a crucial role in regulating immune responses that are essential for tissue repair and bone healing (Li et al., 2022). To investigate the immunomodulatory effects of LA@BC on macrophages, RAW264.7 cells were treated with different experimental groups (Control, BC, LA@BC) for 3 days. Immunofluorescence analysis (Figure 4A) revealed that LA@BC treatment significantly reduced the expression of iNOS (M1 marker, red) and increased the expression of CD206 (M2 marker, green), while DAPI (blue) stained nuclei consistently across all groups. Quantitative analysis demonstrated that LA@BC-treated cells exhibited 30% lower iNOS fluorescence intensity and 1.5-fold higher CD206 fluorescence intensity compared to Control and BC groups, indicating a shift toward M2 polarization (Figures 4B, C). At the transcriptional level, RT-qPCR analysis showed that LA@BC downregulated TNF-α (pro-inflammatory M1 cytokine) by 50% and upregulated IL-10 (an anti-inflammatory M2 cytokine) by 2.5-fold compared to the Control group (Figures 4D, E). ELISA results further confirmed that LA@BC-treated cells secreted significantly lower levels of IL-17 (pro-inflammatory) and higher levels of IL-4 (anti-inflammatory) compared to other groups (Figures 4F, G). These data collectively suggest that LA@BC potently suppresses M1 polarization and promotes M2 activation. The predominance of M2 macrophages can reduce inflammatory damage to the local tissue, thereby contributing to the maintenance of microenvironment homeostasis. Hence, it was expected that the anti-inflammatory properties of the LA probiotics biofilm positively facilitated bone repair, demonstrating its potential as a therapeutic strategy for promoting tissue regeneration *in vivo*.

### 3.5 PL@TCP promoted osseointegration *in vivo*

**FIGURE 5:**
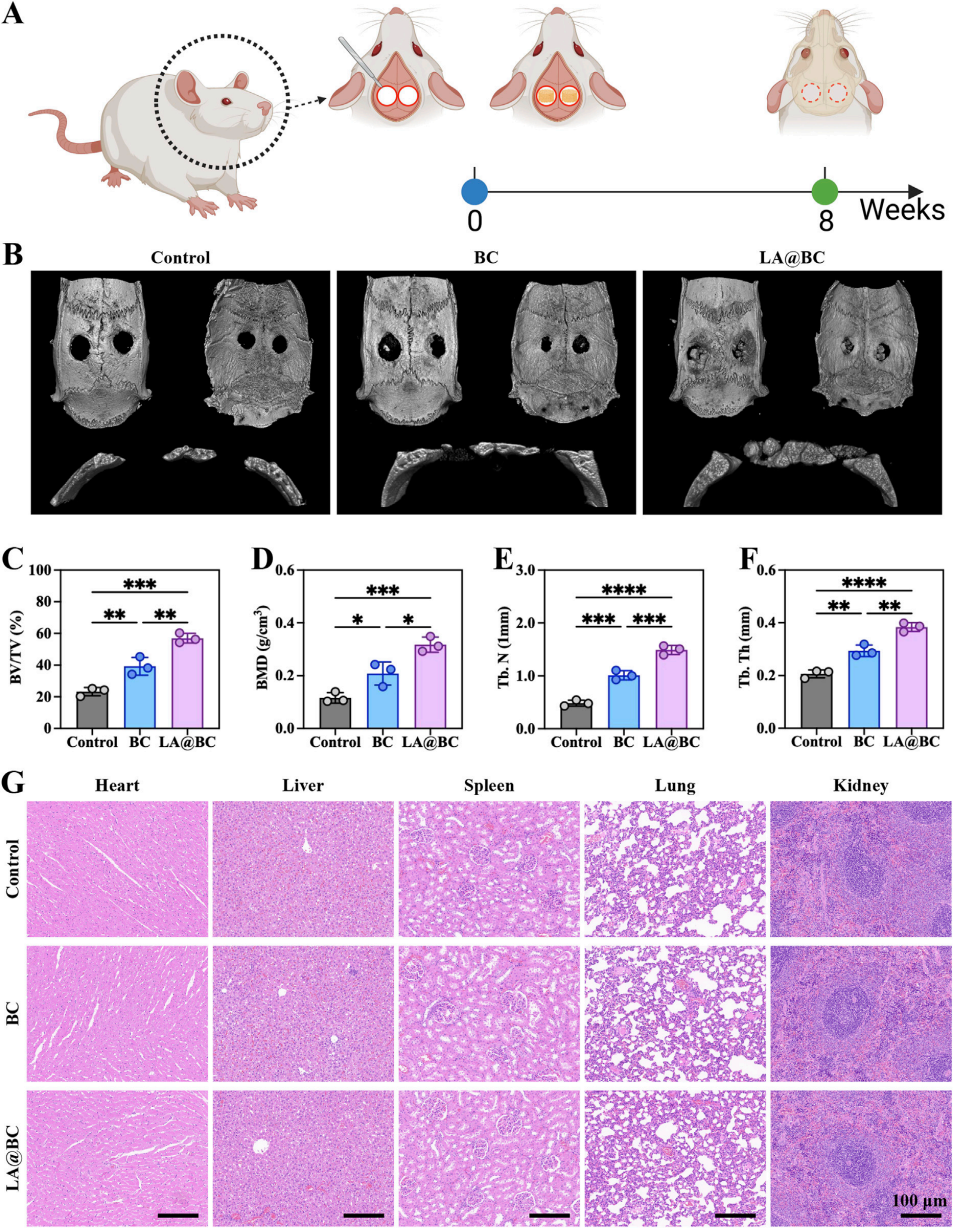
LA@BC improved repair of cranial bone defect *in vivo*. **(A)** Scheme presented the process of bone defect model establishment, scaffold implantation, and osteogenic analysis. **(B)** 3D micro-CT images of cranium at week 8 post-surgery. **(C–F)** Quantitative analysis of osteogenesis-related parameters including BV/TV, BMD, Tb.N and Tb.Th. **(G)** HE staining for heart, liver, spleen, lung and kidney tissues at week 8 post-surgery. *P < 0.05, **P < 0.01, ***P < 0.001, ****P < 0.0001.

A timeline scheme illustrates the experimental workflow, including bone defect induction, scaffold implantation, and osteogenic evaluation over 8 weeks (Figure 5A). Three-dimensional micro-CT images of cranial defects at week 8 revealed that the LA@BC group exhibited substantial bone regeneration, whereas the Control and BC groups displayed residual fibrous tissue or unhealed defects (Figure 5B). Quantitative analysis of osteogenic parameters demonstrated that LA@BC led to superior bone repair. Specifically, the bone volume fraction (BV/TV) increased by approximately 2-fold compared to the Control group (Figure 5C). Additionally, bone mineral density (BMD) reached 0.318 ± 0.017 g/cm^3^ in the LA@BC group, significantly higher than the values observed for the BC (0.208 ± 0.025 g/cm^3^) and Control (0.116 ± 0.011 g/cm^3^) groups (Figure 5D). Furthermore, both trabecular number (Tb.N) and thickness (Tb.Th) increased by approximately 2-fold and 1-fold respectively, in the LA@BC group compared with the Control group, respectively, indicating the formation of a mature trabecular structure (Figures 5E, F). Hematoxylin and eosin (H&E) staining of major organs (heart, liver, spleen, lung, and kidney) at week 8 revealed no pathological damage, confirming the biocompatibility of LA@BC (Figure 5G).

**FIGURE 6:**
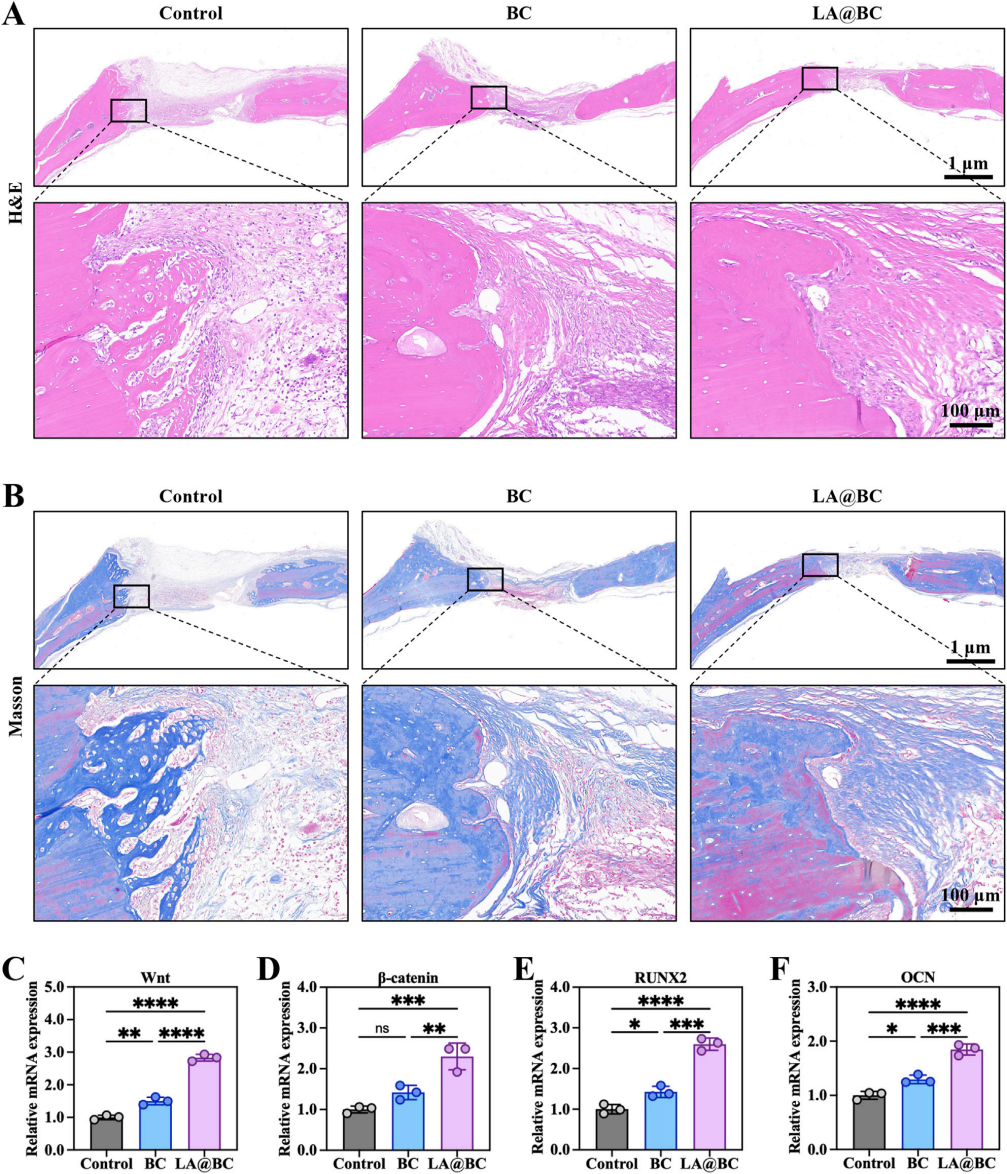
The osteogenic properties of LA@BC *in vivo*. **(A)** H&E staining for bone defects and surrounding normal bones at week 8 post-surgery. **(B)** Masson staining for bone defects and surrounding normal bones at week 8 post-surgery. **(C–F)** Relative mRNA expression of Wnt/β-catenin pathway genes Wnt, β-catenin, RUNX2 and OCN at week 8 post-surgery. *P < 0.05, **P < 0.01, ***P < 0.001, ****P < 0.0001.

Histological evaluation of cranial bone defects at week 8 post-surgery revealed that the LA@BC group exhibited better bone regeneration, characterized by abundant new bone formation and collagen deposition, as shown by Masson staining (orange-red regions) (Figures 6A, B). In contrast, the Control and BC groups displayed incomplete defect healing with predominant fibrous tissue, as illustrated by H&E staining (blue-stained areas) and sparse collagen observed in the Masson staining. High-magnification images confirmed a dense osteoid matrix formation in the defects treated with LA@BC. The porous BC scaffold provides essential mechanical support for new bone growth, while the surface modification of LA@BC enhances cell adhesion and osteoconduction. This combined effect allows for efficient integration of the scaffold with the host bone, facilitating an optimal environment for bone regeneration.

Gene expression analysis further demonstrated that LA@BC significantly upregulated the expression of genes associated with the Wnt/β-catenin signaling pathway, including Wnt, β-catenin, RUNX2, and OCN, when compared to the Control and BC groups (Figures 6C–F). Notably, the expression levels of Wnt, β-catenin, RUNX2 and OCN in the LA@BC group were approximately 2.5-fold, 2-fold, 2.5-fold and 1.5-fold respectively higher than those in the Control group, indicating advanced osteoblast differentiation. This pathway plays a critical role in mesenchymal stem cell commitment to osteoblasts and in promoting bone matrix mineralization.

**FIGURE 7:**
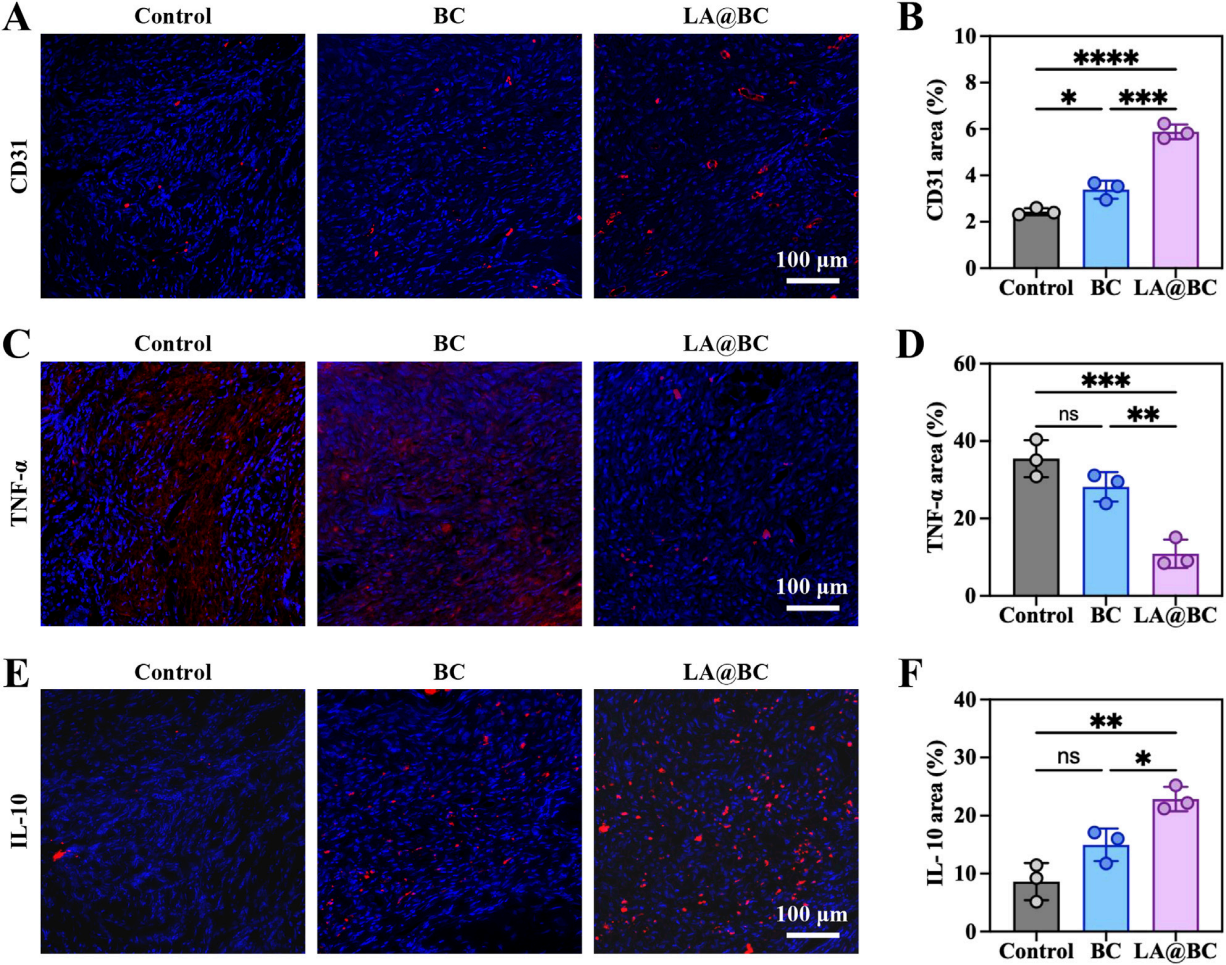
The vascularisation and M2 polarizating properties of LA@BC *in vivo*. **(A)** Immunofluorescent staining of nucleus and CD31 in defect areas at week 8 post-surgery. **(B)** Quantitative fluorescence intensity of CD31. **(C)** Immunofluorescent staining of nucleus and TNF-α in defect areas at week 8 post-surgery. **(D)** Quantitative fluorescence intensity of TNF-α. **(E)** Immunofluorescent staining of nucleus and IL-10 in defect areas at week 8 post-surgery. **(F)** Quantitative fluorescence intensity of IL-10. *P < 0.05, **P < 0.01, ***P < 0.001, ****P < 0.0001.

The effects of LA@BC on angiogenesis and M2 macrophage polarization were assessed in a cranial osteomyelitis model. Immunofluorescence staining of nuclei (blue) and CD31 (red, a marker of endothelial cells) conducted at week 8 post-surgery revealed that the LA@BC group exhibited enhanced vascularization, characterized by dense CD31-positive networks extending into the defect area (Figure 7A). In contrast, the Control and BC groups displayed sparse vasculature. Quantitative analysis confirmed that LA@BC significantly increased CD31 fluorescence intensity by approximately 2.5-fold compared to the Control group, indicating improved blood vessel formation (Figure 7B). The promotion of vascularization by LA@BC establishes a nutrient-rich microenvironment conducive to osteoblast migration and bone matrix deposition. This finding aligns with previous *in vitro* research emphasizing the critical role of angiogenesis in the healing of large bone defects.

Additionally, immunofluorescence staining for TNF-α (red) showed minimal inflammation in the LA@BC group, whereas the Control and BC groups exhibited high levels of TNF-α expression associated with severe inflammatory infiltration (Figure 7C). Quantitative analysis demonstrated that LA@BC reduced TNF-α levels by approximately 70% compared to the Control group, highlighting its anti-inflammatory efficacy (Figure 7D). Immunofluorescence staining for IL-10 (red) revealed a strong M2 macrophage polarization in the LA@BC group, with abundant IL-10-positive cells surrounding the defect area (Figure 7E). Conversely, Control and BC groups showed low or absent IL-10 expression. Quantitative analysis confirmed that the LA@BC group exhibited approximately 2.5-fold higher IL-10 fluorescence intensity compared to the Control group, consistent with M2 activation (Figure 7F). The LA@BC-induced M2 macrophage phenotype, characterized by increased IL-10 and decreased TNF-α, promotes an immunosuppressive effect that attenuates chronic inflammation.

## 4 Discussion

This study presents a novel approach to bone regeneration using the LA@BC scaffold, which integrates the immunomodulatory properties of LA biofilms with the osteoconductive capabilities of calcium phosphate ceramics. Our results showed that LA@BC achieved superior bone repair through a multifaceted mechanism involving the modulation of osteogenesis, angiogenesis, and immune responses, while avoiding the risks associated with live bacterial therapies. The absence of cytotoxicity (Figures 2A, 3A), sustained cell viability (Figures 2B, 3D), and H&E staining of major organs (Figure 5G) confirmed the biocompatibility of LA@BC, consistent with previous reports on the safety of inactivated probiotics in tissue engineering (Tan et al., 2020). The retention of these properties after UV inactivation addressed concerns about infection risk and regulatory hurdles. At the cellular level, LA@BC significantly enhanced the osteogenic differentiation of BMSCs by activating the Wnt/β-catenin signaling pathway. Notably, this was evidenced by the upregulated expression of key osteogenic markers (Wnt, and β-catenin) at both the mRNA levels, as well as increased alkaline phosphatase activity and calcium deposition. The Wnt/β-catenin signaling pathway plays a critical role in osteogenesis by regulating the differentiation, proliferation, and survival of osteoblasts, which are essential for bone formation (Cui et al., 2020). Activation of this pathway occurs when Wnt proteins bind to Frizzled receptors and the co-receptor LRP5/6 on the surface of osteoprogenitor cells, leading to the stabilization and accumulation of β-catenin in the cytoplasm (Xiong et al., 2024). Once stabilized, β-catenin translocates to the nucleus, where it drives the expression of key osteogenic genes such as RUNX2 and osteocalcin, thus promoting osteoblast differentiation and function. In addition to facilitating osteoblast commitment, Wnt/β-catenin signaling enhances osteoblast proliferation and resistance to apoptosis, ensuring an adequate supply of functional osteoblasts for effective bone regeneration (Hu et al., 2018). Our findings indicated that LA probiotic biofilms have the potential to activate this pathway, thereby improving osteogenesis.

In terms of angiogenesis, LA@BC induced a robust proliferation in HUVECs, as reflected by increased migration, and tube formation (Figure 3). Upregulation of VEGF and bFGF further supported the creation of a vascularized microenvironment critical for nutrient delivery and bone mineralization (Ge et al., 2024; Mu et al., 2025). This angiogenic synergy with osteogenesis mirrored the natural coupling of blood vessel invasion and bone formation observed in cranial bone defect repair. In the biological healing process, the formation of new blood vessels, driven by angiogenesis, was crucial for delivering essential nutrients to the regenerating bone tissue. This interplay not only supported the survival and proliferation of osteoblasts, the cells responsible for bone formation but also facilitated the transport of signaling molecules that promote osteogenic differentiation. Therefore, the simultaneous enhancement of angiogenesis and osteogenesis created a conducive microenvironment that was vital for effective bone repair. Importantly, LA@BC outperformed conventional TCP-based scaffolds in both *in vitro* and *in vivo* models, underscoring its dual functionality as a bioactive and structural platform.

The immunomodulatory role of the LA@BC scaffold was another critical finding. The implantation of biomaterials often triggers an inflammatory response within the host tissue, which can significantly impair tissue healing and lead to traumatic ectopic ossification (Li J. et al., 2021; Wang et al., 2023). Initially, the body perceived the implant as a foreign object, leading to the activation of the immune system (Qiao et al., 2021). This activation resulted in the recruitment of inflammatory cells, such as macrophages and neutrophils, to the implantation site. While this inflammatory response was a natural part of the healing process, excessive or prolonged inflammation could impede tissue regeneration and compromise bone healing (Wang et al., 2025). The inflammatory cytokines may interfere with the differentiation and function of osteoblasts, thereby delaying or preventing effective osseointegration (Loi et al., 2016). Consequently, managing the inflammatory response associated with implanted materials was essential to optimize bone healing outcomes and ensure the success of the implant. By shifting macrophages toward an M2 phenotype while suppressing proinflammatory cytokines (Figure 4), LA@BC established an anti-inflammatory microenvironment conducive to bone healing. The anti-inflammatory effect endowed LA probiotics with the potential for application in bone tissue engineering.

In the rat cranial bone defect model, the effects of the LA@BC scaffold on promoting osteogenesis, enhancing angiogenesis, and regulating macrophage polarization were further validated. Micro-CT and histological analyses demonstrated a significantly improved bone regeneration capacity in the LA@BC group. The upregulation of the Wnt/β-catenin signaling pathway further supported this *in vivo* result, consistent with the cellular results. The inflammatory response observed in the LA@BC group was downregulated, reducing chronic inflammation that could impair bone healing. The increased polarization of M2 macrophages, characterized by increased IL-10 expression, reflected a shift toward a healing phenotype. In addition, the increased expression of CD31 and enhanced vascularization were crucial for the functionality of osteoblasts and bone regeneration. These results supported the importance of adopting a multifunctional approach to address the complexities of bone repair, indicating that the incorporation of LA probiotic biofilm into bioceramics was a promising strategy for improving bone healing.

The materials used in the LA@BC scaffold were readily available and could be procured with relative ease. Furthermore, LA, a widely studied probiotic, was commercially produced, ensuring that sufficient quantities were accessible for clinical applications. Probiotic-modified scaffolds were straightforward to manufacture, allowing for reproducible and consistent production. In addition, the results of both *in vitro* and *in vivo* experiments demonstrated their biosafety, which was a crucial prerequisite for clinical application. The accessibility of components, ease of preparation, and favorable safety profile of the LA@BC scaffold collectively supported its operability and clinical feasibility. These considerations made the LA@BC scaffold as a promising candidate for future applications in immunological treatment and bone defect therapy.

Despite the demonstration of the benefits of LA@BC in this study, certain limitations persisted. The long-term stability of inactivated LA biofilms *in vivo* and the precise contribution of LA probiotic composition to signaling remain to be elucidated. Moreover, subsequent research should examine whether LA probiotic biofilms possess an augmented effect on the mechanical strength of bioceramics for load-bearing applications. Nevertheless, the integration of probiotic bioactivity with ceramic scaffolds provided a theoretical basis for the clinical application of biomaterials, which balanced innovation with translational potential.

## 5 Conclusion

In conclusion, LA@BC bioactive ceramic demonstrated significant potential to enhance osteogenic repair and promote vascularization in cranial bone defects. Through the activation of the Wnt/β-catenin signaling pathway and the modulation of M2 macrophage polarization, LA@BC not only accelerated the proliferation and differentiation of bone marrow stem cells but also fostered an anti-inflammatory microenvironment conducive to tissue regeneration. The enhanced vascularization induced by LA@BC treatment resulted in a nutrient-rich microenvironment, which facilitated endothelial migration and bone matrix deposition, both of which are critical for effective healing. Furthermore, the favorable results regarding cytotoxicity and biocompatibility indicate that the inactivated LA probiotic biofilm is a safe therapeutic strategy. These results demonstrate that the LA@BC scaffold is a promising bioactive therapy for bone defects.

## Data Availability

The raw data supporting the conclusion of this article will be made available by the authors, without undue reservation.
